# Transcriptome sequencing of seven deep marine invertebrates

**DOI:** 10.1038/s41597-024-03533-4

**Published:** 2024-06-24

**Authors:** John A. Burns, Joost Daniels, Kaitlyn P. Becker, David Casagrande, Paul Roberts, Eric Orenstein, Daniel M. Vogt, Zhi Ern Teoh, Ryan Wood, Alexander H. Yin, Baptiste Genot, Robert J. Wood, Kakani Katija, Brennan T. Phillips, David F. Gruber

**Affiliations:** 1 Bigelow Laboratory for Ocean Sciences, East Boothbay, ME 04544 USA; 2 Monterey Bay Aquarium Research Institute, Research and Development, Moss Landing, 95039 USA; 3 School of Engineering and Applied Sciences, Harvard University, Cambridge, MA 02138 USA; 4 Department of Ocean Engineering, University of Rhode Island, 215 South Ferry Road, Narragansett, RI 02882 USA; 5 PA Consulting, Concord, MA 01742, USA; 6 Department of Natural Sciences, Baruch College, City University of New York and PhD Program in Biology, CUNY Graduate Center, New York, NY 10010 USA

**Keywords:** RNA sequencing, Evolutionary genetics

## Abstract

We present 4k video and whole transcriptome data for seven deep-sea invertebrate animals collected in the Eastern Pacific Ocean during a research expedition onboard the Schmidt Ocean Institute’s R/V Falkor in August of 2021. The animals include one jellyfish (*Atolla* sp.), three siphonophores (*Apolemia* sp., *Praya* sp., and *Halistemma* sp.), one larvacean (*Bathochordaeus mcnutti*), one tunicate (*Pyrosomatidae* sp.), and one ctenophore (*Lampocteis* sp.). Four of the animals were sequenced with long-read RNA sequencing technology, such that the reads themselves define a reference assembly for those animals. The larvacean tissues were successfully preserved *in situ* and has paired long-read reference data and short read quantitative transcriptomic data for within-specimen analyses of gene expression. Additionally, for three animals we provide quantitative image data, and a 3D model for one siphonophore. The paired image and transcriptomic data can be used for species identification, species description, and reference genetic data for these deep-sea animals.

## Background & Summary

The ocean’s midwater: away from coasts, below the top 200 m of water, and above the ocean floor is the largest contiguous ecosystem on earth, yet, it remains one of the least explored for biodiversity^1^. It is home to a large diversity of pelagic animals that live suspended in a vast volume of water that encompasses over 1 billion km^3^ globally. Many of those animals are deep-sea gelatinous zooplankton (e.g., jellyfish, ctenophores, salps, siphonophores) that are particularly difficult to approach, observe, sample, and preserve owing to their extremely soft tissues^2^.

Gelatinous animals of the midwater harbour novel biology that can be unveiled through 3D modelling and whole genome and transcriptome sequencing^3^. They offer vital ecosystem services, establishing connections to the global food web, fisheries, and carbon sequestration in the deep sea, but estimates from the deep sea are lacking^4^. Deep sea gelatinous animals are underrepresented in genomic and transcriptomic datasets^2,5–8^.

**Fig. 1:**
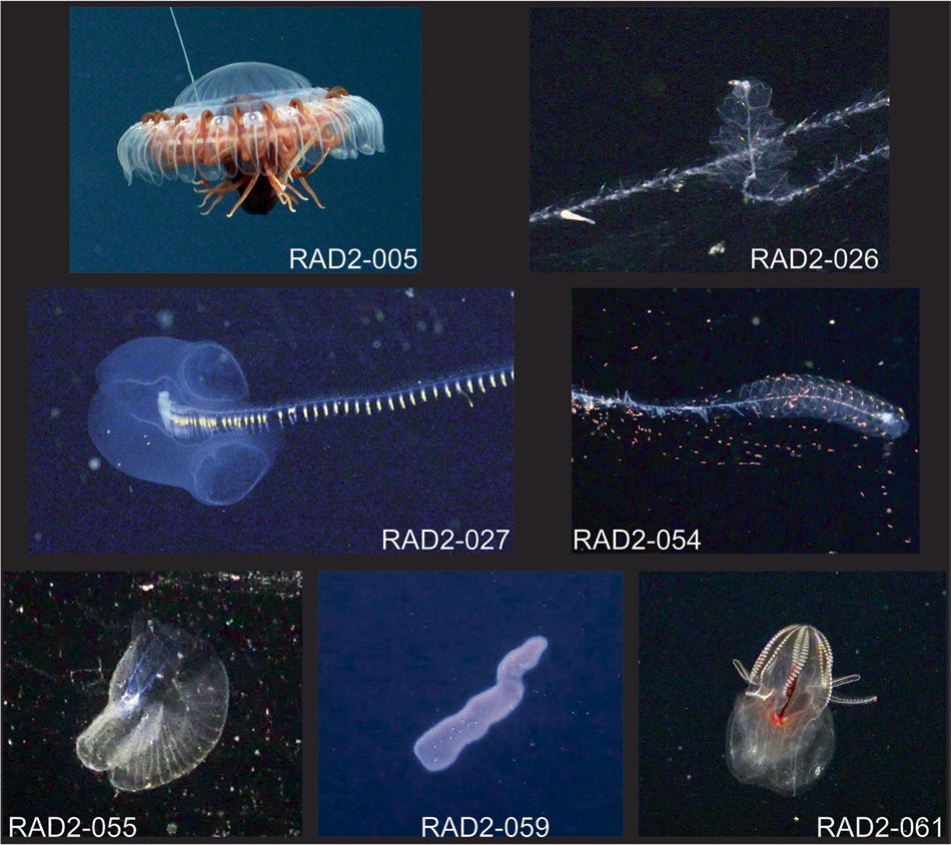
Image captures of the seven animals for which whole transcriptome, video, and image data are presented. The images are *in situ* captures from the 4k camera on the R/V SuBastian. Scale information is not possible on these image captures due to the differences in zoom, distance, and angle from the submarine for each animal. Additional data from the provided video captures can be used to get a sense of scale for each animal.

The combination of whole transcriptomic genetic data coupled with high quality qualitative and quantitative image data allows for in depth exploration of the biology and evolution of deep-sea animals^3^. Here we present image and whole transcriptome data for 7 deep sea gelatinous animals (Fig. 1) that were collected as part of a research project that involved development of new technologies and elaboration of an integrated workflow to capture *in situ* image and genetic data from delicate gelatinous zooplankton^3^. The animals include 3 siphonophores: *Praya* sp., *Apolemia* sp., and *Halistemma* sp.; one jellyfish, *Atolla* sp.; one larvacean, *Bathochordaeus mcnutti*; one tunicate, *Pyrosoma* sp.; and one ctenophore, *Lampocteis* sp. Each dataset adds new information that either enriches available sequence and image data for the group, or adds the first genetic data available for the species.

## Methods

### Sample collection

In August 2021, a ten-day integrated expedition aboard the Schmidt Ocean Institute’s R/V Falkor was conducted on the Pacific Ocean off the coast of San Diego, CA^3^. The expedition involved seven dives of the remotely operated vehicle (ROV) SuBastian, each lasting approximately eight hours. Launches took place in the mid-afternoon local time, with recovery occurring towards midnight to capture the diel migration patterns of ocean animals.

The ROV SuBastian was equipped with three innovative systems for midwater exploration:

1. The Deep Particle Image Velocimetry (DeepPIV) laser imaging system [ref], which allows for quantitative imaging of marine organisms and particles.
2. The EyeRIS plenoptic camera system, which enables 3D imaging and tracking of marine life.
3. The rotary actuated dodecahedron (RAD-2) for specimen encapsulation and tissue sampling^3,9^. RAD-2 is a 12-sided encapsulation device that uses a rotary actuator to open and close. It is constructed from anodized aluminum, stainless steel, and Hydex 301 for corrosion resistance. The central face is instrumented with a tissue sampling device driven by a thruster motor and an inlet port for pumping sampled tissue into a Labs Suspended Particulate Rosette (SuPR) sampling system^10^.

These tools allowed the team to conduct visual identification, quantitative imaging, and targeted sampling of marine organisms during the dives. In the data reported here only one specimen had sufficient imaging data for 3D reconstruction, the siphonophore *Halistemma* sp. as indicated in Table 1. In the companion publication four animals were reported with innovative imaging and digital modeling of each^3^.

**Table 1:** Data types and locations for each specimen.

Specimen ID	Organism type	Species	*In situ* pres.	Library type	SRA ID	4k Video	Advanced imaging
**RAD2_005**	Jellyfish	*Atolla* sp. c JB-2023	No	Illumina PE library	SRX22654762	10.5281/zenodo.10987660	ND
**RAD2_026**	Siphonophore	*Apolemia* sp.	No	Illumina PE library	SRX22659312	10.5281/zenodo.10987828	ND
**RAD2_027**	Siphonophore	*Praya* sp.	No	Illumina PE library	SRX22890523	10.5281/zenodo.10988012	ND
**RAD2_054**	Siphonophore	*Halistemma* sp.	No	Long read assembly-hq-main	SRX22659229	10.5281/zenodo.10988194	https://sketchfab.com/3d-models/deeppiv-3dr-knobby-siphonophore-halistemma-142856c1663c49669cc2c8114ecb0253
**RAD2_054**	Siphonophore	*Halistemma* sp.	Yes	Long reads-lq	SRX22659230	Same as above	Same as above
**RAD2_055**	Larvacean	*Bathochordaeus mcnutti*	Yes	Long read assembly-hq-main	SRX22501790	10.5281/zenodo.10988949	ND
**RAD2_055**	Larvacean	*Bathochordaeus mcnutti*	Yes	Long reads-lq	SRX22501791	Same as above	ND
**RAD2_055**	Larvacean	*Bathochordaeus mcnutti*	Yes	Illumina PE library	SRX23507012	Same as above	ND
**RAD2_055**	Larvacean	*Bathochordaeus mcnutti*	Yes	Illumina PE library	SRX23507013	Same as above	ND
**RAD2_059**	Tunicate	*Pyrosomatidae* sp.	Yes	Long read assembly-hq-main	SRX22659253	10.5281/zenodo.10998462	ND
**RAD2_059**	Tunicate	*Pyrosomatidae* sp.	Yes	Long reads-lq	SRX22659254	Same as above	ND
**RAD2_061**	Ctenophore	*Lampocteis* sp.	Yes	Long read assembly-hq-main	SRX22659251	10.5281/zenodo.10998479	ND
**RAD2_061**	Ctenophore	*Lampocteis* sp.	Yes	Long reads-lq	SRX22659252	Same as above	ND

A shipboard-mounted echosounder system (EK60) was used to locate high biodensity layers in the water column for focused ROV exploration. Once organisms of interest were identified and imaged using the ROV’s onboard 4k video camera and specialized imaging systems, targeted sampling was conducted using the RAD-2 sampler. The RAD-2 sampler collected tissue samples in conjunction with a McLane Labs SuPR Sampler^10^. The SuPR sampler uses a high-flow pump to sample a specified volume of water onto 100μm mesh filters with injection of a preservative (RNALater in this case) *in situ*.

### Sample processing

Post-capture, tissue samples were preserved *in situ* using a custom RNA preservative (described as RNALater) according to the formulation by Malmstrom, 2015^11^ to stabilize RNA for downstream sequencing. The SuPR sampler was preloaded with 10 L of RNALater per dive. After encapsulation and maceration, 0.5–1 L of RNALater was pumped through the filters to replace seawater and preserve the sample, taking on average 3:17 minutes.

Samples not preserved *in situ* were transferred to RNALater upon recovery of the ROV on deck. Large tissue fragments were placed directly into cryotubes with RNALater. If tissue could not be easily separated from filters, the entire filter was placed in a 50 mL Falcon tube with RNALater. Samples were incubated at 4 °C overnight for penetration then transferred to −80 °C. Samples in RNALater were transported to the Bigelow Laboratory for Ocean Sciences in East Boothbay, Maine for further processing. For RNA extraction, 100 mg tissue subsamples were lysed in Trizol reagent. Total RNA was then extracted using spin columns following the manufacturer’s instructions for the TRIzol Plus RNA Purification Kit (Invitrogen, Waltham, MA). RNA integrity was checked using an Agilent TapeStation with RNA Integrity Number (RIN) values reported in Table 2 and TapeStation data files available in a Zenodo data repository^12^.

**Table 2:** RNA and sequence statistics for each specimen.

Specimen ID	TapeStation ID	Total RNA (ng)	RIN	*in situ* preservation?	Sequence Platform Decision	Read Count	Illumina Reads Mean Quality Score	Illumina Reads %bases > Q30	Long read assembly N50
RAD2-005	FK002	4,052	5.4	No	Short Read Only	94,993,602	35.69	92.46	NA
RAD2-026	FK053	781	7.2	No	Short Read Only	92,621,455	34.89	88.56	NA
RAD2-027	FK054	5,332	5	No	Short Read Only	95,381,336	35.47	91.38	NA
RAD2-054	FK104	8,280	8.8	Yes	Long Read Only	48,934 hq (assembly equivalent)	NA	NA	2,109
RAD2-055	FK105	8,700	10	Yes	Long Read and Short Read	Short: 16,174,087 Long: 35,516 hq (assembly equivalent)	35.53 (rep1) 35.56 (rep2)	91.60 (rep1) 91.74 (rep2)	2.363
RAD2-059	FK114	2,221	9.8	Yes	Long Read Only	74,051 hq (assembly equivalent)	NA	NA	2,428
RAD2-061	FK121	409	8.9	Yes	Long Read Only	32,790 hq (assembly equivalent)	NA	NA	2,366

### Sequencing

To generate reference transcriptomes, long-read transcriptome sequencing was conducted using the Pacific Biosciences long read Isoform-Sequencing (Iso-Seq) method. Iso-Seq allows for the sequencing of full-length transcripts, facilitating isoform discovery and annotation. Iso-Seq read files can function as reference assemblies^13^. Functional completeness of the transcriptomes was assessed using BUSCO v5.2.2 against the metazoan database^14^.

Short-read, mRNA sequencing was conducted on the Illumina HiSeq platform for quantitative analysis of samples with high quality RNA, and to recover whole-transcriptome information on samples where the RNA quantity was too low for long read sequencing, or the RIN number indicated degradation such that long read sequencing would likely be unsuccessful (Table 2). Sequencing was conducted at Genewiz (Azenta, Genewiz, South Plainfield, NJ) with the goal of generating high integrity, functionally complete transcriptomic datasets.

## Data Records

For each specimen we have uploaded raw sequence data to the NCBI sequence read archive (SRA)^15^. Those data are whole transcriptome data in the form of short read paired-end.fastq files for Illumina paired end (PE) libraries, and in the form of high quality (hq) and low quality (lq) long read.fasta files for transcriptomes sequenced using the Pacific Biosciences Iso-Seq method. The choice of sequencing platform was guided by RNA quality metrics and the total quantity of RNA recovered from each specimen. The long read-hq files can be used as reference transcriptome assemblies for the specimen without further processing. The short-read data requires de novo transcriptome assembly for full analyses. For the larvacean *Bathochordaeus mcnutti* (specimen ID RAD2_055) we created a long-read reference assembly as well as two short read quantitative datasets, which are technical replicates: sequence data generated from the same RNA pool. The short-read data can be used for quantitative assessment of highly and lowly expressed genes within the animal, and may provide insight into processes like mucus production for the larvacean “house”^16^. Also included are links to 4k video files for each animal and advanced imaging data where available^17–23^.

## Technical Validation

Prior to each sequencing run, the RNA was subject to electrophoretic analysis on an Agilent TapeStation. We saw that specimens that were not preserved *in situ* tended to have lower RNA integrity numbers (RINs) with evidence of RNA degradation. We pursued short read sequencing of those samples to build a reference transcriptome, even from partially degraded RNA. Samples that were successfully preserved with RNALater at depth had higher RIN values and were subject to long-read sequencing. Because of the high RNA quality, high RNA abundance, and uniqueness of the sample, we pursued long and short read sequencing of specimen RAD2-055, from the larvacean *B. mcnutti*. For specimens RAD-005, RAD2-026, and RAD2-027, the read depth is sufficient for de novo assembly of reference transcriptomes for those animals. For RAD2-054, RAD2-055, RAD2-059, and RAD2-061, the long-read transcriptome data can serve as a reference assembly.

### Molecular identification

Additional validation included manual assembly of marker gene sequences from short read datasets by finding an exact match to a marker seed and extending that match forward and backward, and selection of marker gene sequences from long read data to verify the organism identifications. Marker gene sequences can be found in the file

MolecularBarcodeSeqs_forValidation.fasta^12^.

#### Short read file molecular identifications

The COX1 sequence from RAD2-005 has 99.83% sequence identity (587/588 matches, 0 gaps) to *Atolla vanhoeffeni* isolate V3709ss2 COX1 gene, GenBank ID: OM214497.1

The COX1 sequence from RAD2-026 has 92% sequence identity (629/682 matches, 0 gaps) to *Apolemia* sp. BO-2009 voucher Hy100.2.1 COX1 gene, GenBank ID: GQ119954.1^24^

The 18 S sequence from RAD2-027 has 99.78% sequence identity (1783/1787 matches, 0 gaps) to an 18 S barcode from *Praya* sp. AGC-2001, GenBank ID: AF358067.1^25^. By the seed-extension method with the short reads, we could not find a hydrozoan COX1 sequence in the RAD2-027 data. We did uncover a COX1 sequence with a 98.98% sequence identity (676/683 matches, 0 gaps) to the COX1 gene from the krill *Euphausia pacifica* voucher Eu27.12.1, GenBank ID: MT826933.1^26^, which is a presumed prey in this case. Upon full transcriptome assembly, the siphonophore and prey transcriptomes should be carefully disentangled prior to functional analyses.

#### Long read assembly molecular identifications

The closest match to the COX1 gene from specimen RAD2-054 in the NCBI nucleotide database is to a *Physonectae* sp. mitochondrial sequence, with 91% identity (1414/1555, 6 gaps) to GenBank ID: OQ957211.1^5^. We have a tentative identification for that individual as *Halistemma sp*. based on morphology, but more molecular data from the group is needed.

The closest match to the COX1 gene from specimen RAD2-055 in the NCBI nucleotide database is to *Bathochordaeus mcnutti* isolate V3647ss1 COX1 gene, with 99.75% identity (399/400 matches, 0 gaps), GenBank ID: KX599264.1^27^

The closest match to the COX1 gene from specimen RAD2-059 in the NCBI nucleotide database is to *Pyrosomatidae* sp. USNM IZ 1449850 COX1 gene, with 100% identity (658/658 matches, 0 gaps), GenBank ID: OK209615.1.

The closest match to the 18 S gene from specimen RAD2-061 in the NCBI nucleotide database is to the ctenophore *Mnemiopsis leidyi* 18 S ribosomal RNA gene, with 98.31% identity (1921/1954 matches, 10 gaps), GenBank ID: AF293700.1^28^. Although we can identify putative COX1 genes from specimen RAD2-061 by searching the long-read assembly with a protein sequence query (tblastn), there are no close matches in the NCBI nucleotide database to the resultant sequences from RAD2-061.

### Functional completeness

**Fig. 2:**
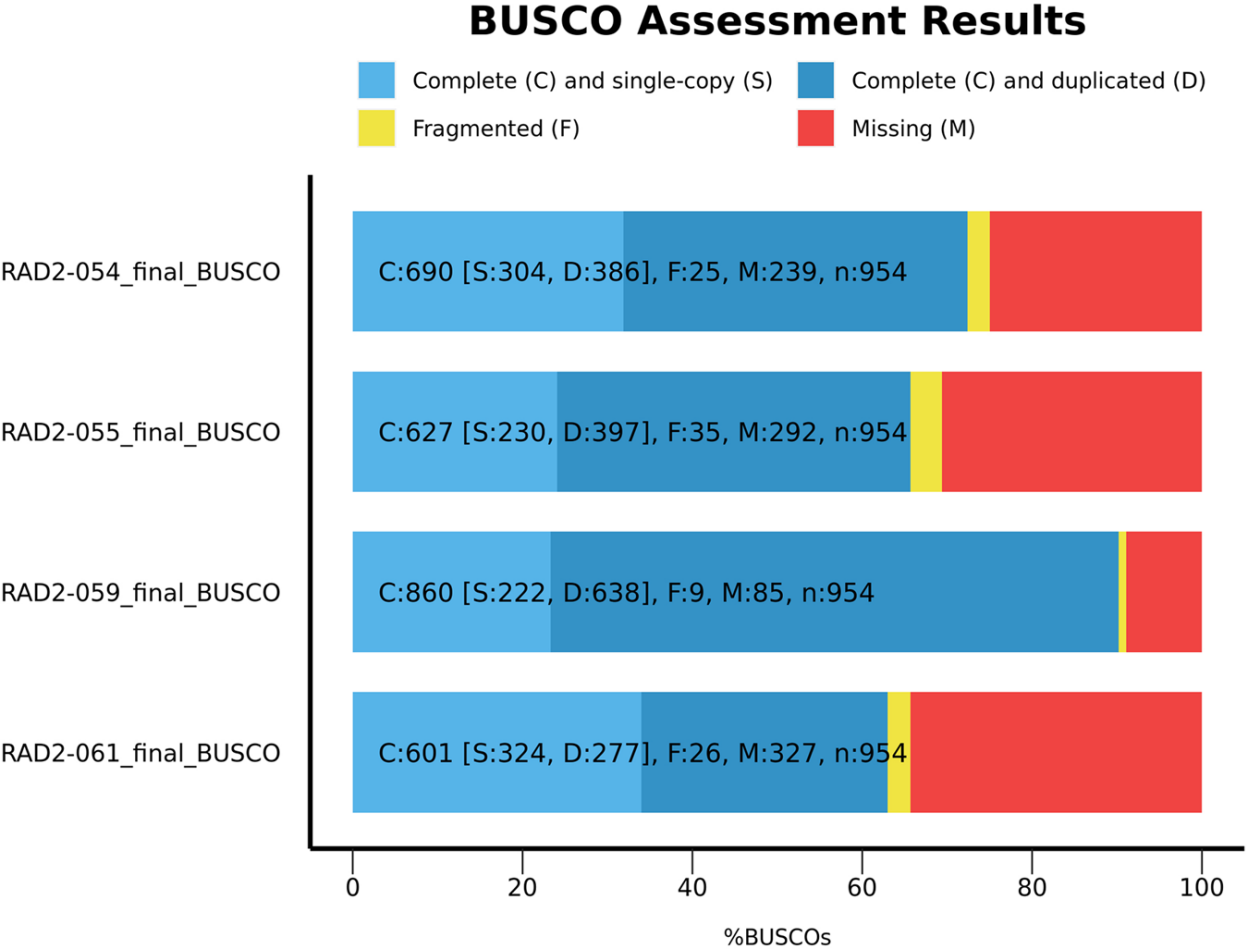
BUSCO completeness metrics for each long-read dataset. Each high-quality long-read sequence file can be treated as a transcriptome assembly. BUSCO was run on each high-quality long-read sequence file using the metazoan BUSCO set to produce these completeness assessments.

For the four long read RNA sequencing datasets, we completed BUSCO analysis^14,29^ to assess functional completeness of the transcriptomic data. We see an average BUSCO completeness of 73% against the metazoan database (Fig. 2), which is similar to completeness scores of the transcriptomes of other deep-sea gelatinous zooplankton^3^.

## Data Availability

No propriety codes were used to generate these data.
